# The troubling story of blood-driven dementias

**DOI:** 10.1038/s41380-018-0225-z

**Published:** 2018-08-20

**Authors:** Marie Rieux, Melanie Alpaugh, Francesca Cicchetti

**Affiliations:** 1 Centre de Recherche du CHU de Québec – Université Laval, Axe Neurosciences, 2705 Boulevard Laurier, Québec, QC G1V 4G2 Canada; 2 Département de Psychiatrie & Neurosciences, Université Laval, Québec, QC G1K 0A6 Canada

**Keywords:** Neuroscience, Diseases

In the mid 1800s, Paul Bert developed a novel model that involved the surgical fusion of two living organisms, which he named parabiosis; a term derived from the English word para (beside) and the Greek word biosis (model of life). Bert was interested in reperfusion of tissue after transplantation. He postulated that blood networks were adaptable and that if two living organisms were joined by the flank, their circulatory systems would correspondingly become coupled. This hypothesis was confirmed when he injected a tracer into the blood of one rat and detected it in the non-injected, but surgically attached animal [1]. The ability of parabiosis to definitively demonstrate the involvement, or lack of involvement, of circulating factors made it a popular technique used to answer a diverse array of research questions ranging from factors influencing obesity, to the cause of dental cavities [2].

Parabiosis has many technical advantages but also presents challenges for the collection of behavioural measures, primarily because of reduced mobility and the inability to separate the performance of one animal from another. Some studies have performed simple motor tests on parabionts, such as the rotarod. However, even if animals are physically capable, scores for each animal cannot be obtained independently [3]. Separation of the animals to facilitate behavioural analysis is also problematic, as the long-term suturing of the joints tends to have longstanding effects on movement. This weakness, in conjunction with tighter ethical regulation of animal experiments, led parabiosis to fall out of favour after 1980. However, a resurgence of this model has occurred in recent years, particularly in the field of neuroscience, where it has been used to demonstrate the detrimental effects of β2-microglublin on the nervous system as well as the benefits of “young blood” on vascular remodelling, neurogenesis, olfactory discrimination and spatial memory in old mice [4–6]. The findings from the young blood studies were sufficiently encouraging to result in Alkahest entering into collaboration with Wyss-Coray to try “the fountain of youth” in a randomized double-blind controlled study of Alzheimer’s disease patients over the age of 50, of which the results are pending.

These studies were important as they demonstrated the ability of peripheral factors to influence the function of the central nervous system, providing proof of principle that parabiosis could be an effective model to resolve an ongoing debate in the field of neurodegeneration; can misfolded proteins initiate disease in an otherwise healthy animal. The study performed by Bu et al. [7]. was the first to use this elegant model to address the potentially pathogenic effect of the presence of misfolded amyloid-β (Aβ) in the blood. Specifically, they used the parabiosis model to connect Aβ transgenic and wild-type (WT) mice and observed that blood-derived Aβ protein can cross the blood-brain-barrier (BBB) and enter the parenchyma of WT mice. Twelve months post surgery, the non-transgenic mice presented with Aβ-related pathologies, deficits of hippocampal neurons and features of angiopathy.

Alzheimer’s disease is an ideal neurodegenerative condition to evaluate the consequences of misfolded protein in the circulation because Aβ is present in peripheral circulation, can cross the BBB and has been shown to have prionic properties in vitro [8, 9]. Therefore, the model was well selected, as were the methods of detection of alterations in the brain. Incorporation of intravital 2-photon microscopy was appropriate for detection of Aβ in the parenchyma and the demonstration of secondary disease markers, such as tau accumulation and vascular pathology, were highly relevant. However, we were most impressed with the clever circumvention of the behavioural limitations of the parabiosis model through the electrophysiological measurement of long-term potentiation as an indication of memory deficits. The delineation of functional changes in the central nervous system in association with the presence of Aβ was a particular strength of this work.

While many aspects of this study were extremely well performed, all studies have limitations. In this work, there are two important limitations to consider. First, Bu and colleagues selected to incorporate non-surgical animals as their controls. In our opinion, this is not ideal as the parabiotic procedure itself has many consequences (inflammation, increased stress) and therefore the potential to activate the immune system due to the presence of foreign blood. These factors undoubtedly affect normal cellular and organ functionality. For these reasons, a more appropriate control would have been WT/WT and Aβ/Aβ parabiotic couples. Second, it would have been very interesting if this study had included conditions where animals were separated after 1 or 2 months and then aged for another 10 months. The authors did demonstrate evidence of seeding by showing the presence of normal mouse Aβ in insoluble aggregates. However, in our opinion, these data could have been further strengthened by separation of the animals to assess aggregation without the constant administration of pathological human Aβ. This experimental paradigm would have been more in keeping with the seeding literature where multiple generations of inoculation are used to demonstrate pathogenicity of misfolded proteins [10].

**Fig. 1:**
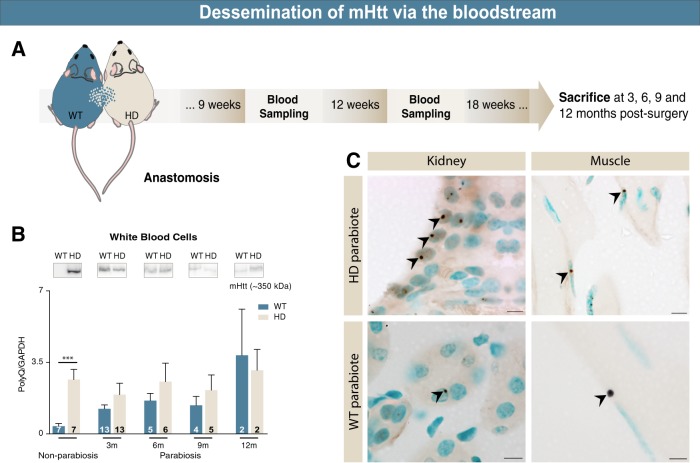
Dissemination of mHtt via the bloodstream. **(a)** WT and zQ175 adult mice underwent parabiosis. Blood sampling was performed every 3 weeks where the various cellular elements were isolated. **(b)** mHtt was detected in white blood cells from WT mice 3, 6, 9 and 12 months post surgery, as well as in platelets and red blood cells (data not shown). **(c)** Post-mortem analyses indicate the presence of mHtt (antibody S829 provided by Gillian Bates; DAB) in several peripheral organs including kidney and muscle tissue in WT mice, 9 months post surgery. Scale bars: 10 µm

The study of Bu et al. is of particular interest to our laboratory as we have published data [11,12], indicating that the toxic protein associated with Huntington’s disease, mutant huntingtin (mHtt), is capable of spreading within the nervous system, inducing a behavioural phenotype in mice lacking the genetic huntingtin mutation. While evocative, these studies were not sufficient to elucidate the mechanism of mHtt spread. Similarly to Bu et al., we have tackled the possibility of mHtt propagation through the blood using parabiosis. To date, our studies suggest that there is transfer of mHtt via the bloodstream at various time points (3, 6, 9 and 12 months) from zQ175 Huntington’s disease to WT mice (Fig. 1a, b) and that there are mHtt aggregates present within the peripheral organs of WT mice paired to zQ175 mice (Fig. 1c).

Both our work and the work of Bu et al. indicate that spreading and induction of neurodegenerative phenotypes is possible. Furthermore, clinical experience illustrates that genetic disorders can be induced in recipients of liver transplants [13]. However, the probability of such an event in the context of Alzheimer’s or Huntington’s diseases appears to be unlikely. In fact, a study looking at more than a million blood transfusion recipients found that there was no evidence of transmission of pathology despite a fairly significant number (2.9%) of transfusions using blood from patients with neurodegenerative diseases [14]. These findings are complementary to the prolonged duration (4 months) of inoculation required to elicit changes in long-term potentiation shown in the work by Bu et al., and supports the theory that the extreme length of the exposure periods required to initiate pathology renders such a phenomenon as highly improbable in the context of blood transfusion. Future work will be required to understand if there is risk associated with organ transplantation from individuals with neurodegenerative disorders, however, for Alzheimer’s disease at least, this possibility seems remote.

These studies also highlight the potential importance of peripheral pathology in neurodegenerative conditions. The emphasis in past research was heavily weighted toward central pathology but it is now becoming more evident that many central disorders are accompanied by peripheral dysfunction. In Huntington’s disease, many peripheral deficits have been described including extreme weight loss [15], muscle wasting [16], cardiac problems [17], changes in hepatic mitochondrial function [18], pancreatic alterations leading to dysregulated glucose homeostasis [19] as well as immune activation [20]. It is important to note that a number of these functions have been shown to correlate with CAG repeat length, cognitive deficits as well as striatal atrophy suggesting that there is an interaction between peripheral and central pathologies. The peripheral disorders reported in the case of Huntington’s disease are not specific to this pathology. Alzheimer’s disease is also accompanied by systemic disorders, such as significant weight loss [21], deregulation of glucose metabolism [22] and the presence of cytokines in the bloodstream, indicating an activation of the immune system [23].

Among the described peripheral alterations, changes in levels of circulating pathological proteins have been described for both Alzheimer’s and Huntington’s diseases. In Alzheimer’s disease, it is theorized that the periphery plays an important part in central protein clearance by degrading and eliminating Aβ [24]. This theory is supported by findings that indicate that peripheral clearance diminishes with age and correlates with Aβ accumulation in the brain [25]. Another study using the parabiosis model provided additional support for this theory by demonstrating that peripheral infusion of blood from WT mice reduced Aβ deposition in the brain of transgenic APP mice [26]. In Huntington’s disease, very little work has been done to adress the metabolism of mHtt in the periphery, but there is one report describing the presence of mHtt in peripheral monocytes with a correlation between the amount of mHtt, disease stage and progression of caudate atrophy [27].

Evidence of a relationship between peripheral protein levels and disease progression suggests that peripheral proteins could be a viable target for therapeutics. One way to tackle this involves the utilization of active and passive immunization, which has previously been tested in Alzheimer’s disease with varying degrees of success. The first immunotherapy trials in Alzheimer’s disease, using active immunization with total Aβ (AN1792) were stopped during phase IIa due to a high occurrence rate of meningo-encephalitis (6%) in patients receiving the vaccine [28]. The exact reasons for this are still not well understood, but it has been speculated that the C-terminus of total Aβ can activate type 2 T-helper cells and consequently cause increased inflammation [29]. More recent trials have moved to passive immunization methods, where antibodies against the protein of interest are injected intravenously. This method avoids the inflammatory complications of active immunization but presents challenges related to low permeability of the BBB to antibodies. Nevertheless, clinical benefits have been observed with this method, suggesting that passive immunization has therapeutic potential in Alzheimer’s disease [30, 31]. In Huntington’s disease, fewer studies have addressed the utility of immunization to target mHtt in either clinical or pre-clinical trials [32]. The ubiquitous expression of mHtt reduces the potential benefits of selectively targeting extracellular toxic protein. Instead, significant research has been focussed on genetic strategies, such as anti-sense oligonucleotides, to reduce the intracellular expression of mHtt [33]. While genetic strategies are directed at preventing the transcription or translation of mHtt, they do not target previously produced the intracellular protein, nor do they neutralize the toxic extracellular protein. The presence of residual mHtt raises the possibility that antibody-based targeting of mHtt may be of great clinical relevance as an adjunct therapy. Combination of antibodies and anti-sense oligonucleotides would permit targeting of intra- and extracellular mHtt, leading to a complete mHtt targeting strategy.

Clinical reports of a role for peripheral pathological proteins, combined with the observations reported by Bu et al. and those from our current work, have important ramifications regarding the consequence of contaminated blood transfusion or tissue transplantation as well as for a wide range of similar disorders of the central nervous system, including the potential elucidation of novel therapeutics.
